# The Benefits of Non-Pharmaceutic Interventions on Intrinsic Capacity in Insulin-Resistant Adult and Geriatric Populations—A Narrative Review

**DOI:** 10.3390/jcm15156101

**Published:** 2026-08-05

**Authors:** Iulia-Daniela Lungu, Adina Carmen Ilie, Ramona Ștefăniu, Sabinne-Marie Albișteanu, Ana-Maria Turcu, Gabriela Grigoraș, Diana-Gabriela Constantinescu, Anca-Iuliana Pîslaru, Ioana Dana Alexa

**Affiliations:** Grigore T. Popa University of Medicine and Pharmacy, 700115 Iași, Romania

**Keywords:** metabolic syndrome, diet, physical exercise, gastrointestinal microbiome, prevention, healthy aging, lifestyle interventions, exercise

## Abstract

Insulin resistance (IR) is a well-established metabolic disorder characterized by reduced responsiveness of peripheral tissues to insulin, leading to hyperglycemia and compensatory hyperinsulinemia. **Background**: In older adults, IR tends to develop gradually and may remain undiagnosed for years due to the insidious nature of the condition, which often lacks overt symptoms. Non-pharmacological interventions refer to the sum of all measures taken in order to improve a certain biological determination or physical parameter and they consist of lifestyle and dietary modifications. Although insulin resistance is recognized as a central mechanism involved in the development of numerous chronic diseases associated with aging, its impact on intrinsic capacity and the mechanisms underlying this relationship are insufficiently synthesized in the literature. In this context, the present narrative review aims to integrate the current evidence on the interaction between insulin resistance and intrinsic capacity, highlighting common biological mechanisms and potential therapeutic and preventive strategies to promote healthy aging. **Methods**: We conducted a literature search in PubMed, Scopus and Web of Science databases, screening the literature published between 2010 and January 2026. The search strategy included keywords and Boolean operators in order to refine the suggestions. Relevant articles, reviews, studies and guidelines were selected based on their relevance to the objective of this review. **Results and Conclusions**: The results of the review highlight that insulin resistance represents a central mechanism contributing to the decline in intrinsic capacity through chronic inflammation, oxidative stress, mitochondrial dysfunction and alteration of the gut microbiota. Current evidence suggests that nutritional interventions, in particular the Mediterranean diet and the DASH diet, can improve insulin sensitivity by modulating the microbiota and reducing systemic inflammation, with the potential to contribute to maintaining functional capacity and promoting healthy aging.

## 1. Introduction

IR is a metabolic disorder characterized by a diminished tissue response to insulin, causing hyperglycemia and compensatory hyperinsulinemia, frequently associated with type 2 diabetes, obesity and metabolic syndrome. In older adults it represents a major risk factor for cardiovascular disease, metabolic disorders and sarcopenia through complex mechanisms that include inflammation, oxidative stress and endothelial dysfunction. With advancing age, insulin sensitivity decreases physiologically due to changes in body composition and reduction in muscle mass and physical activity, as well as increased systemic inflammation, which causes IR to evolve slowly and often remain undiagnosed.

In this context, the concept of intrinsic capacity (IC), defined by the WHO (World Health Organization) as the set of physical and mental capacities of an individual, becomes essential for maintaining functionality in senior populations, being determined by five main components (vitality, cognition, locomotion, and psychosocial and sensory functions), which reach their peak in adulthood and progressively decrease thereafter.

Given the aging of the population, the limitations of pharmacological therapy, the impact of polypharmacy on quality of life and the lack of specific treatments for sarcopenia and vitality decline, non-pharmacological interventions, mainly based on lifestyle and dietary changes, become essential for supporting intrinsic capacity and promoting healthy aging.

Although there is a growing body of evidence supporting the role of IR in the pathogenesis of metabolic and cardiovascular diseases, and the importance of IC as a determinant of healthy aging, the relationship between these two concepts remains poorly understood. It is currently not fully understood how IR influences each of the domains of IC and by which biological mechanisms—including chronic low-grade inflammation, oxidative stress, mitochondrial dysfunction, and alterations in the gut microbiota—it contributes to age-related functional decline. Furthermore, the available evidence is scattered and comes from distinct research areas, limiting an integrated understanding of these interactions. Therefore, a critical synthesis of the literature is needed to clarify the relationship between IR and IC and to highlight potential targets for preventive and therapeutic interventions.

The conceptual hypothesis of this review is that IR represents a central mechanism contributing to the decline in IC through interaction with chronic inflammation, oxidative stress, mitochondrial dysfunction and alterations in the gut microbiota, and that nutritional and lifestyle interventions can modulate these mechanisms, contributing to maintaining functionality and promoting healthy aging.

This narrative review aims to critically summarize and discuss current evidence regarding the interplay between IR and IC, with a particular focus on the underlying pathophysiological mechanisms and the role of nutritional and lifestyle interventions in improving insulin sensitivity, preserving IC, and promoting healthy aging.

## 2. Methods

This narrative review was conducted and reported in accordance with the SANRA (Scale for the Assessment of Narrative Review Articles) recommendations [1]. A literature search was conducted in the PubMed, Scopus, and Web of Science databases, including articles published between January 2010 and January 2026. The search focused on studies investigating the relationships among IR, gut microbiota, IC, aging and lifestyle interventions.

The search strategy included Medical Subject Headings (MeSH), where applicable, and free-text keywords, including: “geriatric population”, “intrinsic capacity”, “ insulin resistance”, “physical exercise”, “diet”, “Mediterranean diet”, “DASH diet”, “gut microbiome”, “frailty” and “cardiovascular disease”, and Boolean operators (AND/OR) to refine the search. To ensure comprehensive coverage of the literature, additional relevant studies were identified through manual screening of the reference lists of key articles and review papers.

Study selection was based on relevance to the objectives of the present review. Original research articles, systematic reviews, meta-analyses, narrative reviews, scoping reviews, and evidence-based guidelines issued by recognized international organizations were considered for inclusion. Publications focusing exclusively on younger populations, studies unrelated to IR, IC or gut microbiota, articles without full-text availability, and publications written in languages other than English were excluded.

The identified records were screened based on title and abstract relevance, followed by full-text review when necessary to confirm their relevance. Given the narrative nature of this review, a formal risk-of-bias assessment was not performed. Instead, emphasis was placed on peer-reviewed publications, landmark studies, highly cited articles, and recent evidence providing substantial contributions to understanding the complex interplay between IR, gut microbiota, IC, and lifestyle-based interventions. The selected literature was narratively synthesized and organized into thematic sections addressing the relationships between insulin resistance, gut microbiota, intrinsic capacity, and lifestyle interventions in older adults.

During the preparation of this manuscript, ChatGPT (OpenAI, San Francisco, CA, USA, GPT-5.5 version was used solely to assist with language refinement and formatting, while DALL·E 3 (OpenAI, San Francisco, CA, USA) was used exclusively to improve the visual style and graphic quality of the figures. The authors have carefully reviewed and edited all content and take full responsibility for the final version of the manuscript.

## 3. Insulin Resistance—Adults Versus Old Persons

### 3.1. Definition and Pathophysiological Mechanisms

IR is a well-established metabolic disorder characterized by reduced responsiveness of peripheral tissues to insulin, leading to hyperglycemia and compensatory hyperinsulinemia. It is commonly associated with type 2 diabetes mellitus (T2DM); IR is also a key feature of several other metabolic abnormalities, such as obesity and metabolic syndrome. In older adults, insulin resistance is a critical health concern, as it is associated with a range of associated or derived pathologies, such as inflammation, cardiovascular disease (CVD), sarcopenia, and neurologic disease. As people age, there is a natural decline in insulin sensitivity. This is partly due to changes in the physiological processes that regulate glucose homeostasis, such as altered fat distribution, reduced muscle mass, and decreased physical activity. Additionally, aging is associated with increased inflammatory markers, oxidative stress, and changes in the autonomic nervous system, all of which can exacerbate insulin resistance. In older adults, IR tends to develop gradually and may remain undiagnosed for years due to the insidious nature of the condition, which often lacks overt symptoms.

Although insulin resistance is recognized as a central mechanism in the development of metabolic disorders, growing evidence suggests that its effects extend beyond glucose homeostasis, influencing essential processes involved in the maintenance of IC, such as muscular functionality, cognitive performance, immune response, and energy metabolism.

The current body of evidence is heterogeneous, as studies vary substantially in terms of the populations examined, the methodologies employed to assess insulin resistance, and the functional outcomes measured. Furthermore, much of the existing research is based on cross-sectional or observational designs, which limits the ability to establish a direct causal relationship between insulin resistance and the progressive decline in IC. In older adults, this association is further confounded by age-related physiological changes, multimorbidity, chronic low-grade inflammation, and lifestyle factors, making it challenging to isolate the independent effect of IR from other biological determinants of aging. Therefore, while IR is increasingly recognized as a potential contributor to functional decline, additional longitudinal studies and rigorously designed interventional trials are required to elucidate its causal role, uncover the underlying mechanisms, and determine whether enhancing insulin sensitivity can effectively preserve IC during aging. Endothelial dysfunction is a prominent link between cardiovascular disease and IR. Aging is often associated with vascular pathology, remodeling, arterial wall thickness, leucocyte adhesion, and atherosclerosis due to endothelial senescence [2]. Advancing age leads to reduced endothelium-dependent dilation (EDD) in response to chemical or mechanical stimuli such as acetylcholine (Ach) or to intravascular shear due to a nitric oxide (NO)-based reduction in oxidative stress states. There is also evidence that ACh-stimulated EDD is decreased with age in coronary arteries, even in adults who do not have major risk factors or clinical disease. Moreover, renal circulation EDD is also affected in the geriatric population and it is a predictor for future renal impairment and decline in glomerular filtration in patients with hypertension [3,4]. Insulin resistance impairs insulin’s ability to stimulate endothelial nitric oxide synthase (eNOS), which plays a crucial role in the vasodilation process. eNOS production is mediated by insulin and vasodilation via phosphatidylinositol 3-kinase (PIK3K) cascade activation. In patients with insulin resistance, the insulin role is shifted towards vasoconstriction, vascular hypertrophy, and atherosclerosis by activation of the mitogen-activated protein kinase (MAPK) pathway [5]. This impairment leads to endothelial dysfunction, a hallmark of early atherosclerosis. Reduced nitric oxide production limits vasodilation, promoting vascular stiffness and increased blood pressure, both of which contribute to CVD. Moreover, obesity and elevated LDL-C levels are also associated with low levels of eNOS expression [6]. By diminishing microvascular perfusion and altering vascular homeostasis, endothelial dysfunction can accelerate the decline of multiple domains of IC, including mobility, vitality, and cognitive function.

Few studies link mitochondrial dysfunction to IR; most of them are preclinical studies targeting mitochondrial defects in animals. IR disrupts the metabolic chain by storing a large amount of fat in the liver and skeletal muscle and affects mitochondrial biogenesis function. Metabolic capacity is reduced in aging populations, as mitochondrial compensating mechanisms tend to diminish [7].

In individuals with IR, there is an imbalance between the production of reactive oxygen species (ROS) and antioxidant defenses, leading to oxidative stress. ROS contribute to endothelial injury, promoting the development of atherosclerosis. In older adults, the cumulative effects of oxidative stress are more pronounced due to diminished antioxidant capacity, further increasing the risk of adverse events. In the context of IR, excessive production of reactive oxygen species amplifies oxidative stress and chronic inflammation, promoting deterioration of muscle, cognitive and vascular function and thus contributing to the progressive decline of IC.

IR links to chronic low-grade inflammation, highlighted by higher levels of proinflammatory cytokines like C-reactive protein (CRP), interleukin-6 (IL-6), and tumor necrosis factor-alpha (TNF-α). These markers can facilitate the formation of atherosclerotic plaques, raising the risk of myocardial infarction and stroke among older adults. Furthermore, the buildup of visceral fat, a recognized factor in insulin resistance, intensifies this inflammatory environment. Obesity and metabolic syndrome (MetS) promote inflammation through activation of pathways such as c-Jun N-terminal kinase (JNK), IκBα kinase, and nuclear factor κB (NF-Κb). These pathways stimulate the production of cytokines, including TNF-α, IL-6, IL-1β, MCP-1, leptin, and resistin, while suppressing adiponectin—a key anti-inflammatory adipokine. These molecules contribute to endothelial dysfunction, impaired glucose and lipid metabolism, and myocardial remodeling [8].

Although each of these factors contributes independently to the development of IR, in clinical practice they frequently coexist and act synergistically. Chronic low-grade inflammation and endothelial dysfunction cause common disturbances in energy metabolism. These mechanisms favor not only the development of IR but also the progressive deterioration of various domains of IC, including mobility, vitality and cognitive function.

### 3.2. Pathophysiological Differences Between Adults and Older Adults

The population aged over 65 years is characterized by an alteration in the organism’s physiological homeostasis, leading to an increased vulnerability to disease due to loss of tissue function and its regenerative capacity. These changes result from systemic, cell-intrinsic and intercellular dysregulations that will be described as follows.

The aging process is associated with complex changes in body composition, including reduced muscle mass, redistribution of adipose tissue to visceral compartments, and decreased mitochondrial function. These changes reduce the ability of peripheral tissues to respond to insulin and promote the establishment of IR even in the absence of overt obesity.

Aging is associated with elevated plasma levels of inflammatory markers, and chronic low-grade inflammation is an essential trigger for atherogenesis, neurodegenerative disease and type 2 diabetes [9]. Inflammaging is a concept defined by low-grade inflammation in an organism with an altered capacity to regulate the components of inflammatory pathways [10].

Another constant that is specific to older age is sarcopenia. It is characterized not only by a reduction in muscle mass, but also by a decline in muscular strength. These changes can be due to several intrinsic factors, such as inflammation, gut microbiota and reduced voluntary neural activation, or extrinsic factors, such as low calorie or, more importantly, protein intake, and physical activity [11].

These pathophysiological changes have direct implications for IC. Sarcopenia and mitochondrial dysfunction lead to reduced mobility and vitality, while systemic inflammation and altered brain metabolism contribute to cognitive and psychological decline. Thus, in the old people, IR should be viewed not only as a metabolic risk factor, but also as an important determinant of the progressive loss of IC.

Trying to evaluate things on a macro level, as people age, there is a change in the cardiovascular system functionality. Seniors have a decreased heart output, which derives from a change in their oxygen arterovenous difference as well as a decrease in their maximum stroke volume and heart rate, which lead to a decline in cardiorespiratory capacity [12].

Although numerous studies support the association between IR and functional decline, the causal relationship remains incompletely elucidated. Most of the evidence comes from observational studies, which limits the establishment of a cause-and-effect relationship. Longitudinal and interventional studies are needed to assess the extent to which improving IR can slow the decline in IC and contribute to promoting healthy aging.

## 4. Intrinsic Capacity

### 4.1. Age-Related Changes in IC

Intrinsic capacity has received growing attention as a fundamental concept in healthy aging, reflecting an individual’s physical and mental capacities that underpin functional ability. It is defined as a composite of physical and mental abilities necessary for an individual to complete everyday tasks successfully. It includes five life domains: vitality, cognition, sensorial capacity, psychological capacity and locomotion [13].

Maintaining optimal physical and mental condition will also ensure seniors are able to carry out daily activities both in their own home and outside it, thus maintaining their independence and autonomy.

Vitality is the element that ties together all the other components of IC. It is defined as the energy regulator, as it mediates the energy-dependent stress response and maintains the homeostasis of the immune and metabolic system. It is assessed by simple tests that evaluate nutritional status as well as blood tests to detect nutritional deficiencies such as hypovitaminosis, anemia, and hypoproteinemia.

Energy expenditure differs during the course of life. There are three types of energy: available energy (total energy), essential energy (the energy necessary for daily activities and life maintenance at rest), and energetic reserve or potential energy (the energy required to maintain independence under stress). With increasing age, the levels of total energy tend to decrease by half [14]. Due to external and internal stressors, seniors have decreased energy reserves, leading to a low need for ATP production, which leads to a decrease in oxidative activity in the myocardium and striated muscle tissue. It has also been demonstrated that in aging adults, both the number and functionality of mitochondria are reduced. This results in a lack of energy, leading to higher levels of ROS [15]. Oxidative stress induces malfunctions in other organs and systems and exacerbates organ pathology.

Mobility is essential for a senior person’s independence. Locomotor capacity is a critical determinant for healthy aging, as its decline is correlated with disability, institutionalization, hospital admissions, and mortality [16].

Sarcopenia is a definite characteristic when referring to the aging population. Aging leads to structural changes in muscular tissue by inverting the proportion of muscle mass to adipose tissue, increasing fibrosis, or shifting the electrical properties of the muscle. It is important to assess muscular function by gait speed or muscle strength rather than muscle mass as strength and performance decline faster than muscle mass [17]. There are numerous studies that have correlated age with a decrease in gait speed [14].

Sensorial capacity refers primarily to visual and hearing capacities, which can be easily assessed by simple screening tools recommended by the WHO [13]. Sensory impairments are highly associated with decreased capacity to perform daily activities, lower mobility and higher incidence of depression [18].

The most studied sensorial impairments are visual and auditory decline, followed by perceptual impairments (taste, smell, touch). As people age, not only do their senses decline, but they also present with polypharmacy that may have adverse effects on taste. Cognitive impairment leads to ineffective sensorial stimulation and malnutrition [19].

Psychological capacity is a broad notion that encompasses emotional functions, personal characteristics and concepts of positive psychology. WHO guidelines emphasize depressive symptoms and their assessment as a primary care point for the aging adults, but the other dimensions need to be taken into consideration for a holistic evaluation [20].

Cognitive decline is not only a neurologic issue, but it is a constant determination in geriatric assessment. It is suggested by symptoms such as loss of focus and attention, loss of short-term memory and reduced ability to solve problems. The importance of cognitive decline screening stems from the fact that in mild to moderate cases, the decline can be reversed by proper training [21]. Birchenough et al. conducted a study on 731 older adults and demonstrated that low IC is associated with a higher risk of developing mild cognitive impairment, which emphasizes the interconnection between all the components of IC [22].

### 4.2. Impact of IR on IC

There is increasing evidence that the incidence of IR increases as people age, and IR is not only considered a simple metabolic finding, but has been identified as a major risk factor for multiple age-related comorbidities due to altered lipid metabolism, metabolic status, endothelial dysfunction, and prothrombotic status.

An article published in the *American Journal of Physiology* demonstrated that the expression of endothelin 1, a vasoconstrictor protein produced by the endothelium, is more prominent in endothelial cells from older men compared to those from younger men. The study also indicated that neither eNOS protein nor its activation is reduced in vascular endothelial cells from older men. Together, these findings highlight the imbalance between vasomodulatory factors with advancing age [23].

IR determines abnormalities in mitochondrial function that are associated with IR and type 2 diabetes.

Excessive intake of nutrients, including of free fatty acids (FFAs) or conditions of prolonged hyperglycemia, leads to increased production of ROS and reduced mitochondrial biogenesis, causing mitochondrial dysfunction. Mitochondrial dysfunction causes decreased β-oxidation and ATP production, as well as increased ROS production, which results in insulin resistance, diabetes, and cardiovascular disease.

These mechanisms do not act independently, but mutually potentiate each other, generating a pathological vicious circle that explains the systemic influence of insulin resistance and its contribution to the global decline in intrinsic capacity, beyond simple metabolic impairment.

A notable gap in the current literature is the absence of longitudinal studies specifically evaluating the impact of IR on IC as a multidimensional construct. Existing longitudinal evidence has primarily focused on the association between IR and individual components of IC, such as cognitive performance, physical function, and muscle strength. Consequently, although these studies support the hypothesis that IR may contribute to IC decline, direct evidence integrating both concepts remains limited, highlighting an important area for future research.

The brain is particularly sensitive to harmful free radical activity due to increased oxygen consumption, limited antioxidant mechanisms, and high levels of polyunsaturated fatty acids (PUFAs). In brains affected by IR, overproduction of ROS leads to oxidative damage associated with ATP depletion, activation of proinflammatory cytokines, accumulation of protein aggregates, and neuronal apoptosis. This causes decreased glucose utilization in the brain, impairs insulin signaling in essential structures such as the hippocampus, and promotes the accumulation of beta-amyloid. Through these mechanisms, insulin resistance is implicated in the pathogenesis of cognitive decline and is correlated with an increased risk of developing dementia [24].

A relevant example is the longitudinal CAIDE (Cardiovascular Risk Factors, Aging and Dementia) study, which followed participants for seven years and assessed insulin resistance by the HOMA-IR index at baseline, as well as cognitive function at reassessment. The results showed that increased HOMA-IR values were associated with significantly poorer cognitive performance at follow-up, suggesting that insulin resistance may contribute to cognitive decline with age [25].

IR negatively influences the locomotor component of intrinsic capacity through its effects on muscle metabolism. This causes a reduction in muscle protein synthesis and an increase in protein degradation, and favors fatty infiltration of muscle tissue. The consequence of these processes is the appearance of sarcopenia, characterized by a decrease in muscle mass and strength, with a direct impact on mobility and an increased risk of falls in geriatric population [26]. Furthermore, in old people with type 2 diabetes, decline in intrinsic capacity is significantly associated with sarcopenia [27].

Further evidence on the impact of IR on components of IC comes from the Baltimore Longitudinal Study of Aging (BLSA). In this longitudinal cohort, elevated HOMA-IR values were associated with reduced skeletal muscle mitochondrial oxidative capacity and impaired muscle performance, independent of other metabolic risk factors. These results suggest that IR affects energy metabolism and muscle function from an early stage, contributing to decreased mobility and vitality, two essential domains of IC [28].

By affecting mitochondrial function and energy metabolism, insulin resistance causes reduced energy production at the cellular level, increased fatigue, and the appearance of exercise intolerance. These changes are reflected early on in the vitality domain, which is often one of the first components of intrinsic capacity to be affected in this context [29].

Insulin resistance is also associated with impairment of the psychosocial domain of intrinsic capacity, being correlated with an increased prevalence of depression, decreased motivation and a tendency towards social isolation, the latter frequently occurring secondary to functional limitations [30].

Regarding sensory functions, although less investigated, there is evidence that insulin resistance may contribute to their deterioration through vascular and neurological mechanisms. Thus, microangiopathy associated with metabolic impairment may negatively influence visual and auditory acuity, and peripheral neuropathy may alter sensory perception, further contributing to the global decline in intrinsic capacity [31,32].

## 5. Non-Pharmacological Interventions in Insulin Resistance

Non-pharmacological interventions are the mainstays of IR management and healthy aging, acting through complementary biological and functional mechanisms. To highlight the role of each strategy and how it contributes to maintaining IC, this section is organized into three main components: physical activity, nutritional interventions, and lifestyle modifications. Physical activity primarily influences insulin sensitivity, muscle function, and physical performance; nutritional interventions modulate metabolic status, inflammation, and gut microbiota composition; and lifestyle modifications, such as optimizing sleep and reducing sedentary behavior, complement these effects by improving metabolic homeostasis and systemic function. Although each intervention exerts specific effects, maximum benefits are obtained through an integrated approach, in which the strategies act synergistically to maintain and improve different domains of IC.

**Figure 1:**
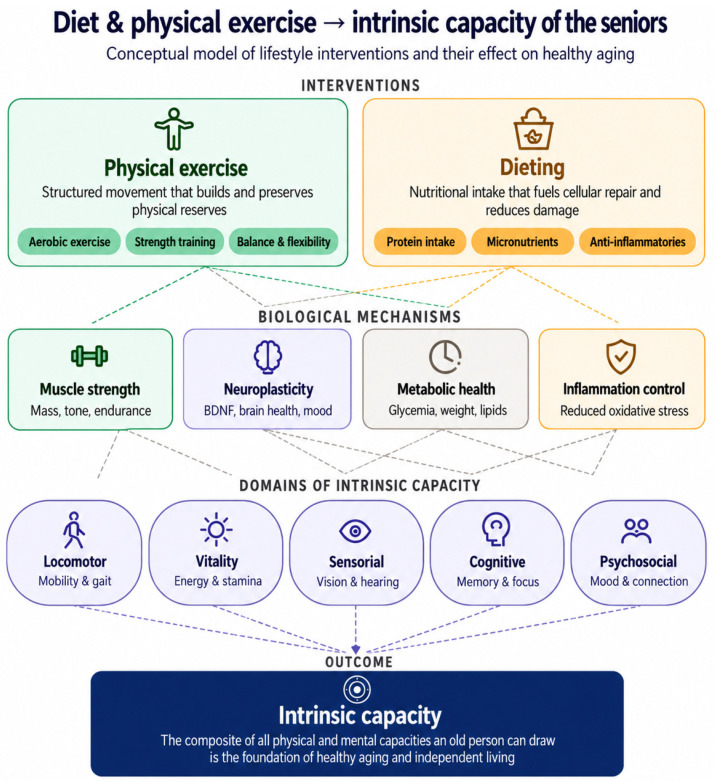
Non-pharmacological interventions in insulin resistance to improve intrinsic capacity.

The cornerstone of managing IR is lifestyle modification, including regular physical activity, a balanced diet, and weight management (Figure 1). Exercise improves insulin sensitivity, reduces visceral fat, and enhances cardiovascular health. Dietary interventions that emphasize a reduction in refined sugars and unhealthy fats can also mitigate the effects of IR on the cardiovascular system. However, a concerning issue in these interventions is sustainability, as several study follow-ups show that nearly half of the patients partaking in an extensive lifestyle program may return to their initial levels of physical activity and diet within 1–5 years post-treatment [33].

### 5.1. Physical Activity

Aging is an important determinant of muscle mass loss, declined strength performance and low cardiorespiratory fitness, all leading to impaired functional capacity. Older individuals often present with varying degrees of physical deconditioning and any kind of physical activity could potentially impair their mental and physical equilibrium.

Exercise training is demonstrated to improve aerobic capacity (AC), quantified by maximum oxygen consumption, leading to increased muscular performance and accelerated metabolic processes. However, AC does not depend only on an individual’s physical activity, but it is also influenced by genotype and the aging process. There are studies that directly link cardiorespiratory fitness to mortality risk in an inverse relationship [34].

Sedentarism is associated with a negative impact on mitochondrial function. Aerobic exercise is demonstrated to stimulate muscular vascularization and increase oxygen inflow to the muscle mitochondria, which have a stimulatory effect on oxidative capacity [35].

A longitudinal, multicenter, randomized trial that followed participants with sedentary behavior between 70 and 89 years over 2.6 years stated that moderate-intensity aerobic exercise reduced major mobility impairment [36].

The brain is also extremely sensitive to aging, as there are many demonstrated age-related changes in white matter. The hippocampus is a brain area that shrinks in late adulthood, leading to a deterioration in cognitive function; the anterior hippocampus has a main role in the acquisition of spatial memory. Aerobic exercise increases brain vascularization and perfusion of key cerebral sites such as the anterior hippocampus, and it increases brain white matter volume in the prefrontal cortex. A study that included 120 participants demonstrated that the more sustained the training, the more beneficial effects were on hippocampal volume and spatial memory [37].

The prevalence of sarcopenia is expected to rise as the population continuously ages. It is associated with an increased risk of falls, functional impairment and increased mortality. Strength training has as its primary goal muscle cell hypertrophy and has been widely studied in healthy older people with a focus on preventing sarcopenia. In recent years, the focus has shifted so older adults with manifested sarcopenia have been included in studies, though the results are contradictory.

In their meta-analysis that included 561 healthy older adults, Chen et al. stated that resistance training has beneficial effects on indices such as body fat mass, handgrip strength, knee extension strength, gait speed and the timed up and go test and no effects on skeletal muscle mass, leg lean mass or appendicular skeletal muscle mass [38]. It still remains unclear which type of exercise or what amount of time is necessary for alleviating sarcopenia in older adults with different diagnoses.

Rodríguez et al. conducted a meta-analysis on 11 RCTs that included inactive adults and older adults, and concluded that even brief bouts of exercise (<5 min, performed at least twice daily for more than three times a week and for over two concomitant weeks) can improve cardiorespiratory fitness, but there is no data described on sarcopenia or other components of functional capacity [39].

In order to optimize resistance training prescriptions, it is important to identify and compare the effects that different exercise intensities and durations have on geriatric populations with sarcopenia. Yan et al. [40] determined that progressively higher weekly training doses (frequency) improved handgrip strength, with a maximum beneficial intensity of 1220 METs-min/week. More frequent training proved to be counterproductive [40]. Load is another important factor to take into consideration, and it is known that high-load resistance training maximizes neuromuscular adaptations and improves muscle strength, even in older and frail adults, but it is more difficult to implement and to sustain in senior populations with comorbidities [41]. Besides load, training volume has an important contribution to muscle hypertrophy. Research has shown that a minimum of 10 sets per muscle group per week is recommended. Another approach showed that moderate training volume (about 24 repetitions per session) was more effective than lower or higher volumes regarding improvements in muscle strength [42,43]. Previous studies showed that slow-velocity training is not as effective in improving muscle strength and function in older adults as high-speed resistance training is, as functional capacity relies on the individual’s adaptability and fast response to environmental changes [44]. Although constant resistance training has a better effect on increasing muscle strength, Walker et al. demonstrated that variable resistance training enhances fatigue resistance, an important asset in older adults’ daily functional tasks, reducing the risk of falls [43]. Institutionalization is an important parameter to consider while studying the senior population, as these patients are seldom in poorer general condition, have a higher rate of cognitive and physical impairment and a more advanced degree of sarcopenia. Yan et al. concluded that non-institutionalized participants had better functional outcomes (timed up and go test) from resistance training, but adults receiving long-term institutional care developed greater knee extension strength [40].

In conclusion, resistance training has a beneficial effect on many components of functional capacity, such as sarcopenia, activities of daily living, and vitality, but training prescriptions should be adapted to initial functional status and dynamically adjusted based on adverse effects, individual tolerance, and resilience.

Concurrent training is a combination of strength and endurance training that has gained interest recently, as it has been demonstrated that seniors need to be part of a program that not only preserves muscle mass, but also improves their strength in order to maintain their independence. VIVIFRAIL is a multicomponent physical exercise program that combines both aerobic and strength components which has proven to have positive effects on functional capacity of older adults [45].

Yuxia Ma et al. [46] conducted a study that included older patients with type 2 diabetes mellitus and sarcopenia who were followed for 12 weeks and stated that concurrent training led to improved grip strength and appendicular skeletal muscle mass index. Moreover, plasmatic biomarker levels related to sarcopenia, such as irisin and 25-hydroxyvitamin D, increased in patients following the intervention [46].

A recently published study performed by Ding et al. investigated the effects of different exercise interventions on lipid profiles in adults with MetS. They concluded that combined aerobic and resistance exercise and high-intensity interval training at moderate doses (500/600–1000 METs-min/week) were efficient in reducing plasmatic cholesterol fractions, thus reducing cardiovascular risk, but also that mind–body exercise (considered as a low-impact intervention) could be a feasible alternative [47].

A meta-analysis published in November 2025 assessing the impact of exercise on sleep quality in older people stated that any type of exercise improved sleep quality, but the most efficient was combined aerobic and resistance training. It also emphasized that the greatest benefit was at around 660 to 990 METs per minute/week [48].

Although all forms of exercise contribute to improving insulin sensitivity and maintaining IC, each modality has specific advantages and limitations. Aerobic exercise is particularly effective in optimizing cardiovascular function, carbohydrate metabolism, and vitality, but has a lower impact on increasing muscle mass and strength compared with resistance training. The latter is the intervention of choice for preventing and treating sarcopenia and for maintaining mobility and functional independence, although the benefits on cardiorespiratory fitness are less pronounced. In contrast, combined programs, which integrate aerobic and resistance exercise, provide the most consistent benefits in multiple domains of IC, including mobility, vitality, and cognitive function, and are recommended as the optimal strategy for most older adults with IR. However, the choice of exercise type should be individualized according to the patient’s age, degree of frailty, comorbidities, functional capacity, and preferences, to maximize adherence and long-term clinical benefits (Table 1).

### 5.2. Nutritional Interventions

Obesity has become a major global health issue and there are international efforts to combat its clinical implications, such as IR. Pharmaceutical agents that have been recently introduced are prohibitive, have a list of contraindications, have secondary effects and are seldom insufficient as monotherapy for treating obesity and for reducing the associated risks. In light of these constraints, there is a need for a more complex strategy in order to treat IR. Functional foods or bioactive compounds are a new approach that are increasingly studied.

MedDiet is a highly studied diet that contains low-glycemic-index foods that reduce postprandial blood glucose fluctuations. It consists of plant-based foods (vegetables, legumes, whole grains, fruits and nuts), uses olive oil as the main fat source, includes fish and poultry for protein intake and limits the consumption of processed foods and red meat. Estruch et al. [49] investigated, in a multicenter randomized trial that enrolled 7447 participants, the effect of MedDiet on CVD primary prevention. They concluded that MedDiet was associated with a 30% CVD risk reduction [49]. Moreover, MedDiet improves hemodynamic homeostasis, improving endothelium-dependent vasodilation, decreases vascular inflammation and LDL-C deposition alongside vascular wall and atherosclerotic plaque formation [50].

Hydroxythyrosol (HT) is a phenolic compound found in olive oil, a main part of MedDiet that has anti-inflammatory, antioxidant, metabolic regulatory and insulin-sensitizing properties. A Chinese study developed on a diet-induced obesity mouse model demonstrated that HT modulates the formation of adipose tissue by targeting the STING1/NLRP3 axis that suppresses adipogenesis. Moreover, it had a significant role in improving glucose homeostasis, lipid metabolism and liver and skeletal muscle function [51].

Dietary soluble or insoluble fiber are other key components of MedDiet that have synergistic and pleiotropic effects on metabolic disorders. Soluble dietary fiber reduces postprandial glucose fluctuations by forming a gel inside the stomach that delays digestion and absorption of carbohydrates. It also improves insulin sensitivity by acting on GLUT4 expression. By binding to bile acids, soluble fiber also impairs lipid absorption, decreasing plasma levels of LDL-C [49]. Insoluble dietary fibers have an important role in satiety by increasing chewing time and acting on the vagus nerve, thus reducing calorie intake and IR [52].

MedDiet is a plant-based diet, which contains important quantities of vitamins such as vitamins A, C, D and E. The antioxidant capacity of both vitamins C and E is known to reduce pancreatic beta cell damage induced by oxidative stress, promoting insulin secretion and sensitivity [53].

MedDiet is rich in bioactive compounds that contribute to a reduction in chronic low-grade inflammation and oxidative stress, two mechanisms involved in the alteration of insulin signaling pathways. Replacing saturated fats with monounsaturated fatty acids, mainly from extra virgin olive oil, improves the fluidity of cell membranes and the functioning of the insulin receptor, facilitating glucose uptake in peripheral tissues.

The beneficial effects of MedDiet on insulin resistance are also mediated by its impact on intestinal microbiota. Numerous studies have shown that increased adherence to this dietary pattern is associated with greater microbial diversity and increased abundance of SCFA-producing bacteria, such as *Faecalibacterium prausnitzii*, *Roseburia* spp., *Bifidobacterium* and *Lactobacillus*. SCFAs, especially butyrate, contribute to maintaining the integrity of the intestinal barrier, reduce intestinal permeability and metabolic endotoxemia, and stimulate the secretion of incretin peptides, such as GLP-1, thereby promoting improved insulin sensitivity. Consistent with these mechanisms, clinical studies have shown that individuals following MedDiet have lower basal insulin and HOMA-IR values, as well as superior glycemic control compared to other dietary patterns.

The DASH diet was initially introduced as an efficient non-pharmacological means to reduce elevated blood pressure values. It consists of a reduction in dietary sodium, red meats, sweets, and food rich in cholesterol and saturated fat, and supplementation of nuts, whole grains, vegetables and low-fat dairy products, providing a higher amount of nutrients such as protein and fiber, but also potassium, calcium and magnesium [54]. Alongside its efficiency in blood pressure control, the DASH diet has a proven beneficial impact on IR, MetS, inflammation and lipid profile by decreasing both systolic and diastolic blood pressure and LDL cholesterol values [55].

Recent studies have demonstrated that adherence to the DASH diet is not only efficient for blood pressure control, but also has beneficial effects on reducing IR and ameliorating lipid and inflammatory profiles. Due to its high contents of dietary fiber, potassium, magnesium, calcium, and antioxidant compounds, the DASH diet contributes to improving carbohydrate metabolism and insulin sensitivity. At the same time, reducing the consumption of saturated fats and refined carbohydrates limits ectopic lipid accumulation and systemic inflammation, favoring the optimal functioning of insulin signaling pathways.

The benefits of the DASH diet on insulin resistance also appear to be mediated by favorable changes in the intestinal microbiota. The increased intake of fermentable fiber stimulates the growth of bacteria producing short-chain fatty acids, especially butyrate, a metabolite involved in maintaining the integrity of the intestinal barrier, reducing systemic inflammation, and improving glucose homeostasis. Interventional studies and meta-analyses have demonstrated that adherence to the DASH diet is associated with decreased basal insulin levels, reduced HOMA-IR index, and improved glycemic control, especially in people with obesity, metabolic syndrome, hypertension, or T2DM. These results support the inclusion of the DASH diet among effective nutritional strategies for the prevention and management of insulin resistance.

A study that included 780 Chinese residents concluded that higher adherence to the DASH diet was associated with lower frailty prevalence [56]. These statements are supported by a US investigation that included 71.941 older women, which described the same results [57].

The association between hypertension and ischemic stroke is well known, implying that higher adherence to the DASH diet reduces the risk of ischemic stroke [58].

Although both MedDiet and the DASH diet have been shown to be effective in improving IR and reducing the risk of cardiometabolic complications, there are important differences between the two dietary patterns. MedDiet has the strongest evidence for improving insulin sensitivity, its effects being attributed mainly to the high content of monounsaturated fatty acids from extra virgin olive oil, polyphenols and omega-3 fatty acids, which modulate inflammation, oxidative stress and the composition of the gut microbiota. In contrast, the DASH diet is characterized by an increased intake of fiber, potassium, magnesium and calcium and a more pronounced restriction of sodium, which makes it especially recommended for people with IR associated with hypertension or metabolic syndrome. However, the implementation of both diets may encounter difficulties related to long-term adherence, the cost of specific foods and the need to change eating habits. Furthermore, the metabolic response may vary depending on individual characteristics, such as gut microbiota profile, genetic predisposition, degree of obesity, and presence of other comorbidities. Therefore, the choice between the MedDiet and the DASH diet should be individualized, taking into account the patient’s clinical and metabolic profile and food preferences, and the possibility of long-term maintenance of the nutritional intervention.

### 5.3. Additional Nutritional Interventions

There is some evidence that suggests that caloric restriction may improve health and lifespan due to activation of cellular damage disposal mechanisms [59,60]. Constant consumption of high-glycemic-index foods can lead to IR, and fiber-rich, low-glycemic-index diets contribute to longer-lasting satiety and better glycemic control.

Carbohydrates are demonstrated to be implicated in the development of obesity, insulin resistance, diabetes and CVD. The “carbohydrate–insulin model” refers to the endocrine dysregulation that a high amount of dietary carbohydrate brings along.

The very-low-calorie ketogenic diet (VLCKD) is a restrictive diet (500–800 calories/day) that includes a fat intake of 15–30 g/day, low carbohydrate content (<50 g/day) and 1–1.5 g of protein/kg of ideal body weight. A 2021 meta-analysis investigating the effects of VLCKD in a European population stated that it is efficient in short-, intermediate- and long-term weight loss and also in improving glycemic and lipid profiles and body composition, preserving muscle mass, strength and resting metabolic rate [61].

On the other hand, Rajaguru et al. published a retrospective study with a focus on 9617 Korean adults and demonstrated that carbohydrate quantity alone was not statistically correlated to a higher MetS incidence, suggesting that population-specific metabolic characteristics may play an important role in these findings [62].

Low-sodium and high-potassium diets, such as MedDiet, have an important role in maintaining healthy blood pressure values by reducing renal sodium reabsorption [63].

Contemporary diets that are rich in meat and lactates induce a hyperacidic environment, leading to increased oxidative stress and inflammation. Mansouri et al. [64] stated that the alkalizing effect of bicarbonate-rich mineral water can reduce the subchronic metabolic acidosis induced by Western diets. This improves insulin receptor binding and reduces insulin resistance, leading to better glycemic control. Moreover, consumption of bicarbonate-rich mineral water is reported to improve lipid burden by decreasing total cholesterol, low-density lipoprotein cholesterol and triglyceride levels [64].

### 5.4. Intestinal Microbiota

The gut microbiota refers to the multitude of microorganisms that are present in the gastrointestinal tract. It has reached researchers’ attentions as a key mediator that links cardiometabolic, endocrine, neural and immune health with diet and physical exercise through the microbiota–gut–brain axis (MGBA).

Diet has a primordial role in maintaining and promoting a healthy microbiota. Dysbiosis, linked to an increased cardiovascular risk, is exacerbated by high-fat diets, whereas high-fiber and plant-based diets favor microbial diversity. There is evidence that even maternal diet has an impact on future offspring’s gut microbiota by increasing the FBR—Firmicutes-to-Bacteroidetes ratio. An increased FBR is correlated with hypertension, obesity and IR development risk [65].

The importance of adherence to MedDiet has been previously discussed and it is important to reiterate that the components of this diet are beneficial for the gut microbiota as well. High adherence is linked toan abundance of health-associated species like *Bacteroides* and *Paraprevotella*, as fiber and healthy fat-rich olive oil determine a favorable microbiota composition, increasing the number of SCFA (short-chain fatty acid)-producing taxa [66].

Polyphenols, which are a key component in MedDiet, remain unabsorbed in high proportions along the gastrointestinal tract and they accumulate in the large colon, where they are metabolized by the intestinal microbiota. This process regulates gut microbiota composition, increasing the number of beneficial probiotics such as *Lactiplantibacillus* spp. and *Bifidobacterium* spp. and decreasing the prevlanence of bacteria associated negatively with human health, such as Clostridium and Fusobacterium species [67].

Similar to MedDiet, the DASH diet has a positive impact on the gut microbiome. Diao et al. [68] conducted a study that included 120 adults with obesity and compared the changes that a low-calorie DASH diet versus a generic low-calorie diet had on gut bacterial taxa. They concluded that participants from the DASH diet group had lower values of plasma trimethylamine N-oxide (TMAO), which is a predictor for cardiovascular mortality and early neurological deterioration [68].

Physical exercise has beneficial effects on cardiorespiratory fitness and IR and it has been demonstrated that it also influences gut microbiota composition by increasing microbial diversity.

### 5.5. Lifestyle Modifications

Sleep disorders in the aging population are very common and represent a major health problem, as they affect quality of life and increase the risk of other diseases. Aging is associated with structural and functional alterations in sleep, including reduced deep sleep, increased sleep fragmentation, and circadian phase advancement. Furthermore, sleep disorders are associated with significant health consequences, including cognitive deterioration, impaired daytime functioning, and increased risk of cardiovascular disease and affective disorders [69].

A 2017 study using objective sleep assessments (polygraphy/polysomnography) and metabolic markers to analyze the association between the severity of sleep-disordered breathing and metabolic dysfunction highlights that insulin resistance is significantly associated with parameters characteristic of obstructive sleep apnea, such as apnea–hypopnea index (AHI), decreased oxygen saturation, and increased frequency of microarousals.

Also, changes in sleep architecture, especially increased light sleep (stage I) and sleep fragmentation, are more pronounced in patients with insulin resistance, suggesting a link between metabolic dysfunction and sleep quality. In contrast, the analysis does not identify significant associations between objective sleep parameters and age-adjusted IGF-1 levels, except for a limited correlation between IGF-1 and subjective sleepiness assessed by the Epworth scale [70].

Skeletal muscle is a major regulatory organ; sarcopenia is a key link between sedentarism and age-related chronic disease. Both acute and chronic inactivity have a negative impact on the aging process. In active people, the aging process is much slower and smoother. For example, the hardening of the arteries and loss of insulin sensitivity, considered “normal” in older people that tend to be sedentary, are greatly reduced or even absent in older people who perform regular aerobic exercise.

Although there is an inevitable decline in maximum heart rate with age (which exercise cannot completely stop), older athletes maintain a VO_2_ max that is much higher than that of young people with sedentary behavior. This “physiological reserve” protects them against disability and premature death [71].

Lack of physical activity may lead to higher levels of inflammatory and immune mediators that are positively associated with aging [72]. Moreover, physical exercise is associated with an improved immune response to the influenza and pneumococcal vaccines [73,74].

Sedentary behavior and low cardiorespiratory function result in depressive symptoms in both men and women, and obesity is positively associated with major depression [75].

To sum up, sleep, sedentary behavior, and gut microbiota are interdependent factors that influence IR and synergistically contribute to the maintenance or decline of IC. Through their effects on systemic inflammation, metabolic homeostasis, neuromuscular function, and cognitive health, these factors simultaneously modulate multiple domains of IC, including cognition, mobility, vitality, and psychological state, highlighting the need for integrated lifestyle interventions to promote healthy aging.

## 6. Impact of Non-Pharmacological Interventions on Functional Capacity

IR is considered to precede the onset of T2DM by 10 to 15 years. IR’s consequence is a lack of glucose disposal in peripheral tissues, especially in skeletal muscle. In a hypercaloric diet, there is an increased necessity for insulin secretion in order to cope with the consecutive states of excess glucose, which leads to a state of hyperinsulinemia. This continuous imbalance between the necessity and the production of insulin will result in exhaustion of beta-2 pancreatic cells, leading to a lower amount of insulin being produced and consequently to the development of T2DM in the long run. Moreover, insulin’s anabolic effect decreases as peripheral IR rises, resulting in weight gain [76]. IR progression can also lead to metabolic syndrome, MASLD (Metabolic Dysfunction-Associated Steatotic Liver Disease) and PCOS (polycystic ovary syndrome), recently proposed to be renamed PMOS (Polyendocrine Metabolic Ovarian Syndrome) [77].

**Figure 2:**
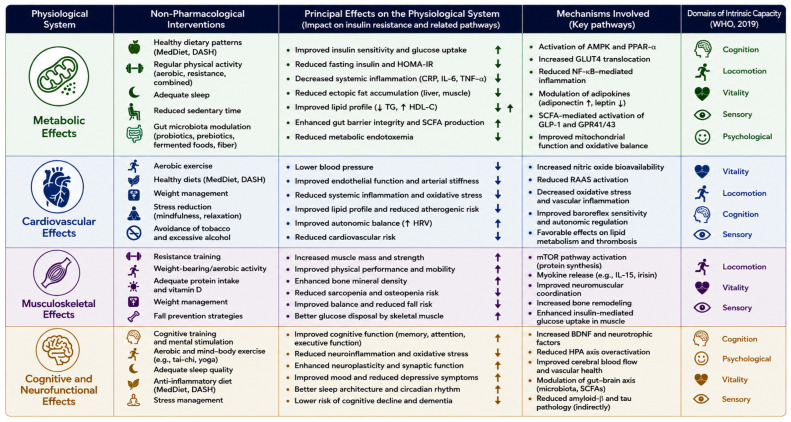
Role of Non-Pharmacological Interventions in Preserving Functional Capacity.

Physical exercise is inversely associated with weight gain and obesity (Figure 2). The ACLS Study (Aerobics Center Longitudinal Study) developed in Texas is one of the most representative studies in its field, which stated that there is a dose–response relationship between energy consumption by aerobic activity and weight loss [78].

Insulin receptors are present in various tissues throughout the body. Although most research on insulin signaling has focused on tissues involved in systemic metabolic regulation, such as adipose tissue, skeletal muscle, the liver, and the brain, it also plays a significant role in other organs, including the heart. A relatively new concept to emerge in the literature is the insulin–heart axis. This refers to both the direct actions that insulin, as a hormone with anabolic properties, has on the myocardium, causing hypertrophy, as well as the indirect actions it has by modulating metabolism and generating the substrate necessary for ATP formation. A disruption in insulin signaling leads to the dysfunctions presented above, determining a proinflammatory, oxidative, and dysmetabolic status that leads to secondary cardiac damage [79].

Moderate- to high-intensity aerobic training has many beneficial effects, amongst which is cardiovascular risk reduction. Spending an additional 1000 kcal per week reduces coronary heart disease burden and additional physical activity of 2000 to 3000 kcal per week is demonstrated to reduce stroke, coronary heart disease and hypertension incidence [80].

Sarcopenia is defined as muscle mass loss or muscle strength loss related to aging. Being an important component of the locomotory system, sarcopenia is associated with disability, functional decline, and frailty in older patients. Besides its role in locomotion, skeletal muscle is the principal organ implicated in insulin-mediated glucose disposal (Figure 2). Epidemiological studies have shown that IR has an important impact on skeletal muscle fiber atrophy, but they have also stated that mitochondrial dysfunction leading to oxidative stress plays its part. Geriatric patients tend to have a lower capacity for producing energy as well as an impaired ability to properly use stored energy due to a reduction in mitochondrial number in persons over the age of 50. This decline is better evidenced in older T2DM patients [35]. Another element associated with IR and sarcopenia is lipid infiltration into muscles, defined as myosteatosis—diagnosed by computerized tomography attenuation coefficient. Studies link the amount of myosteatosis with the onset of IR, and also with physical performance and muscle strength in seniors [81].

The HealthABC study demonstrated a strong relationship between T2DM and muscle mass loss in older adults, alongside the InCHINATI study, which found that older age is associated with a higher incidence of IR, the latter being an independent determinant of weaker muscle strength [82,83].

The brain contains insulin receptors, and their expression is elevated in the hippocampus and the CA1 field, which is responsible for spatial memory training. Insulin receptor number declines with age, and increasing evidence is gathering in favor of linking Alzheimer’s disease (AD) with a reduced number of insulin receptors, naming AD as “type 3 diabetes” [84].

Evidence suggests that insulin administration may significantly influence learning outcomes, as demonstrated in experimental research conducted on both mice and humans. Intravenous administration or intranasal application of insulin has been shown to enhance memory capabilities in healthy individuals as well as in patients experiencing memory impairments [85]. IR was also associated with impaired verbal memory due to reduced hippocampal volume in geriatric patients [86]. Higher HOMA-IR results were related to poorer outcomes in cognition and psychomotor speed in a study that followed 269 dementia-free patients aged 65–79 for 7 years [24].

Moreover, IR promotes an inflammatory milieu, which leads to microglial activation. Inflammatory cytokines such as IL-1β and IL-6 interfere with hippocampal synaptic plasticity. The brain’s antioxidant capacity declines with age and the accumulation of inflammatory cytokines and of advanced glycation products are associated with a higher risk of developing AD [87].

Stroke is the third biggest cause of disability and the second biggest cause of mortality globally. Long-term observational studies stated that adherence to the DASH diet is associated with efficient blood pressure reduction and a better control over body weight, leading to a subsequent reduction in the risk of ischemic stroke [58].

Nguyen et al. investigated the impact of vitamin supplementation in the prevention of stroke. It stated that vitamin C may reduce ischemic stroke risk and vitamins B6 and B12 and folic acid supplementation may reduce hemorrhagic stroke and transient ischemic stroke in adults [88].

## 7. Comparison Between Adults and Older Adults with Insulin Resistance

A sedentary lifestyle is a constant in the aging population and it is a combined result of multiple factors, such as: sarcopenia, cognitive impairment, depression and low vitality. The synthesized evidence indicates that combined programs (aerobic + resistance) are the most effective in the long term, leading to simultaneous improvements in insulin secretion and associated metabolic markers (Table 2). At the mechanistic level, the benefits of exercise are explained by multiple biological pathways: increased expression of the GLUT4 transporter in muscle, improved mitochondrial function, reduction in visceral and ectopic adipose tissue, and reduction in chronic low-grade inflammation. In addition, physical activity can support the secretory function of pancreatic β cells. Zhang Q et al. [89] emphasize the importance of the dose–response relationship (intensity, frequency, and duration), suggesting that the effectiveness of interventions depends on the individualized adaptation of exercise programs for the geriatric population. At the same time, the need for large-scale clinical trials to clarify mechanisms and optimize exercise prescriptions is highlighted [89].

Cadore et al. reported that concurrent training leads to lower power gains and strength in older people, compared to strength training alone. This phenomenon is known as “the interference effect.” [12].

Malnutrition and obesity are components that increase the risk of morbidity and mortality; however, these issues are rarely a focus when talking about geriatric prevention.

Vieira-Lara et al. [90] highlight that both aging and a high-fat diet cause a significant reduction in peripheral and hepatic insulin sensitivity, with these changes preceding a decline in mitochondrial lipid oxidation capacity. The study also demonstrates that aerobic (voluntary) exercise improves insulin sensitivity, but its effectiveness is dependent on the nutritional context. More specifically, under conditions of a balanced diet (low-fat diet), physical activity increases insulin sensitivity at the muscle level concomitantly with an improvement in the capacity to oxidize fatty acids. In addition, physical exercise prevents the age-related decline in hepatic insulin sensitivity. In contrast, in the context of a high-fat diet, these beneficial effects are diminished or absent, suggesting a critical interdependence between diet and the metabolic response to exercise. The study highlights the existence of specific tissue adaptations (muscle versus liver) and emphasizes that the response to lifestyle interventions is not uniform, but varies according to age and diet composition. Thus, aging amplifies the susceptibility to diet-induced metabolic dysfunctions, but these effects can be partially counteracted by adequate physical activity, especially in the context of a healthy diet [90].

In seniors, there are pathophysiologic changes that alter the components of functional capacity. There is an increase in total body fat and a decrease in fat-free mass [91]. These changes are accelerated by intrinsic determinants of hypercatabolism, such as intercurrent diseases, as well as extrinsic factors that will be discussed further on. Sex is another important factor to consider, as women are more prone to malnutrition than men [92].

Affective alterations are seldom found in geriatric assessment. They lead to altered taste and a lower activity status, determining malnutrition over time [93]. In order to maintain their independence, a senior person has to be able to go shopping and prepare and to cook their meals. These functions are limited in institutionalized persons, leading to a higher rate of depression and malnutrition amongst the non-community-dwelling geriatric population [92].

There is a bidirectional relationship between malnutrition and sarcopenia. A frail person, with decreased muscular tissue, has a greater risk of falls and a lower tolerance to performing physical exercise, leading to a lower accessibility for supermarkets or local shops if they are located at a greater distance. This implies that access to fresh food is limited and that they consume fewer vitamins and are more prone to malnutrition. At the same time, if an aging person does not have access to sources of protein, their muscle mass declines [94].

## 8. Conclusions

Insulin resistance is emerging as a central determinant of intrinsic capacity decline in the geriatric population, going beyond the scope of a simple metabolic disorder and involving multiple interconnected pathophysiological mechanisms, such as chronic low-grade inflammation, oxidative stress, mitochondrial dysfunction and impaired endothelial function. Through its systemic action, it simultaneously contributes to the deterioration of several domains of intrinsic capacity, including cognition, locomotion, vitality, psychosocial components and sensory functions.

In this context, non-pharmacological interventions, especially those focused on lifestyle modification, physical activity, and nutritional optimization, play an essential role in preventing and ameliorating the effects of insulin resistance on intrinsic capacity. Integrating these strategies into clinical practice, along with the use of the concept of intrinsic capacity as a functional assessment tool, may contribute to promoting healthy aging and maintaining autonomy and quality of life in senior populations.

Current evidence suggests that non-pharmacological interventions are essential for the prevention and management of insulin resistance and for maintaining IC in older adults. Exercise programs that combine aerobic and resistance training, along with MedDiet, have the strongest evidence for improving insulin sensitivity, preserving muscle and cognitive function, and supporting healthy aging. The DASH diet and personalized strategies to control energy intake and macronutrient composition have favorable effects, but the evidence is less extensive and more varied. Interventions to modulate gut microbiota, optimize sleep, and reduce sedentary behavior are promising but require further validation in high-quality longitudinal and clinical studies. Understanding biological mechanisms, identifying specific biomarkers, and developing personalized interventions will be essential to optimize prevention and treatment strategies dedicated to maintaining IC and promoting healthy aging.

From a clinical perspective, the management of insulin resistance should be based on a personalized and multidimensional approach that takes into account the patient’s age, IC, comorbidities, nutritional status, and FC. Instead of uniform lifestyle recommendations, interventions should be tailored to the metabolic and functional profile of each individual by individualizing the prescription of physical exercise, optimizing nutritional interventions, promoting quality sleep, and reducing sedentary behavior. In the case of older adults, therapeutic goals should go beyond simply improving insulin sensitivity, while also aiming to maintain muscle mass, mobility, cognitive function, and other domains of IC. From a public health perspective, early identification of IR and implementation of multidisciplinary lifestyle interventions throughout the life course can contribute to reducing the burden of chronic diseases associated with aging, preventing functional decline, and promoting healthy aging. In this context, integrating routine assessment of IC into clinical practice may facilitate early identification of individuals at risk and the implementation of individualized preventive and therapeutic strategies, with benefits to long-term prognosis and quality of life.

Despite significant progress in understanding the relationship between IR, gut microbiota, and IC, many gaps remain in the literature. Most evidence comes from observational or short-term interventional studies, which limits the establishment of causal relationships and the assessment of long-term effects. Longitudinal studies and randomized clinical trials are needed to investigate how multidimensional lifestyle interventions, including exercise, nutritional interventions, and gut microbiota modulation, simultaneously influence IR and domains of IC. Future research should also aim to identify biomarkers capable of reflecting early changes in IC and facilitate the development of personalized prevention and treatment strategies tailored to the metabolic and functional profile of each individual.

## Figures and Tables

**Table 1:** Effects of different exercise modalities on insulin resistance and intrinsic capacity: physiological mechanisms, clinical benefits, and practical recommendations.

Exercise Modality	Principal Benefits in Insulin Resistance	Main Physiological Mechanisms	Practical Recommendations for Older Adults	Effect on IC Domains
Aerobic exercise	Improves insulin sensitivity, glycemic control, cardiorespiratory fitness, endothelial function, body composition and cardiovascular health; may contribute to preserving mobility and vitality	Increases GLUT-4 translocation and glucose uptake, enhances mitochondrial biogenesis, improves lipid oxidation, reduces systemic inflammation and oxidative stress, increases skeletal muscle perfusion	≥150 min/week of moderate-intensity aerobic activity (or 75 min vigorous), performed 3–5 days/week; walking, cycling, swimming and dancing are appropriate options	vitality, locomotion, cognition
Resistance training	Increases muscle mass and strength, improves insulin sensitivity, reduces sarcopenia risk, enhances functional independence and mobility	Stimulates muscle protein synthesis, activates insulin signaling pathways, increases glucose storage capacity, improves mitochondrial function and resting metabolic rate	2–3 sessions/week involving all major muscle groups; 1–3 sets of 8–12 repetitions with progressive overload according to functional capacity	locomotion, vitality
Combined exercise	Produces greater improvements in glycemic control, insulin sensitivity, physical performance and body composition than either modality alone; supports several domains of intrinsic capacity simultaneously	Combines metabolic adaptations induced by aerobic exercise with increases in muscle mass and strength induced by resistance training; reduces inflammation and improves metabolic flexibility	Combination of aerobic and resistance training throughout the week, individualized according to age, frailty and comorbidities	locomotion, vitality, cognition, psychological well-being
Age-specific adaptations	Improves adherence, reduces fall risk, preserves functional capacity, quality of life and independence	Individualized progression minimizes injury risk while optimizing neuromuscular adaptations, balance and metabolic responses	Exercise prescription should consider frailty status, cognitive function, comorbidities and baseline physical capacity; balance and flexibility exercises should be incorporated regularly, particularly in frail older adults	all domains through improved adherence and safety

**Table 2:** Age-Related Differences in Insulin Resistance: Adults and Older Adults.

Aspect	Adults	Older Adults	Clinical Implications for Intrinsic Capacity
Insulin sensitivity	Greater improvement following lifestyle interventions due to preserved metabolic flexibility	Improvement occurs but is often attenuated because of anabolic resistance, chronic low-grade inflammation, and multimorbidity	Early intervention essential to preserve metabolic health and delay decline in intrinsic capacity
Response to aerobic exercise	Significant improvements in insulin sensitivity, cardiorespiratory fitness, and body composition	Similar benefits, although smaller improvements may occur due to reduced aerobic capacity and functional limitations	Supports mobility, vitality, cardiovascular health, and endurance
Response to resistance exercise	Increases muscle mass and strength effectively	Particularly important because it counteracts sarcopenia, improves muscle quality, and enhances glucose uptake despite anabolic resistance	Preserves mobility, vitality, and independence, and reduces frailty risk
Combined exercise training	Optimizes metabolic control and cardiovascular fitness	Produces the greatest overall functional benefits by simultaneously improving strength, balance, endurance, and metabolic regulation	Positively influences multiple intrinsic capacity domains simultaneously
Nutritional interventions	Mediterranean and DASH diets effectively improve insulin sensitivity and reduce metabolic risk	Similar benefits, but adequate protein intake, micronutrients, and individualized nutritional support become increasingly important because of malnutrition risk and altered nutrient metabolism	Supports vitality, cognition, muscle function, and healthy aging
Gut microbiota modulation	Greater microbial adaptability following dietary changes	Age-related dysbiosis may reduce responsiveness, although increased fiber intake, probiotics, and prebiotics remain beneficial	Improves metabolic homeostasis, immune regulation, and cognitive health
Weight loss strategies	Moderate caloric restriction generally preserves lean mass when combined with exercise	Excessive caloric restriction may accelerate muscle loss and functional decline if not combined with resistance training and adequate protein intake	Weight management should prioritize preservation of muscle mass and physical function
Sedentary behavior reduction	Rapid metabolic improvements after increasing physical activity	Even modest reductions in sedentary time improve physical performance, glucose metabolism, and functional independence	Helps maintain mobility and delays disability
Sleep optimization	Improves glucose metabolism and hormonal regulation	Substantial benefits, although sleep disorders are more prevalent and often multifactorial	Supports cognition, psychological well-being, metabolic regulation, and vitality
Overall response to multimodal lifestyle interventions	High responsiveness with substantial metabolic improvements	Benefits remain clinically significant but require individualized, multidomain interventions addressing frailty, comorbidities, nutrition, and physical capacity	Integrated interventions best preserve all domains of intrinsic capacity and promote healthy aging

## Data Availability

No new data were created or analyzed in this study. Data sharing is not applicable to this article.

## Abbreviations

The following abbreviations are used in this manuscript:

25(OH)D	25-Hydroxyvitamin D
AC	Aerobic Capacity
ACh	Acetylcholine
AD	Alzheimer’s Disease
AHI	Apnea–Hypopnea Index
CRP	C-Reactive Protein
CVD	Cardiovascular Disease
DASH	Dietary Approaches To Stop Hypertension
EDD	Endothelium-Dependent Dilation
eNOS	Endothelial Nitric Oxide Synthase
FBR	Firmicutes-To-Bacteroidetes Ratio
FFAs	Free Fatty Acids
HT	Hydroxythyrosol
IC	Intrinsic Capacity
IL-6	Interleukin-6
IR	Insulin Resistance
JNK	C-Jun N-Terminal Kinase
MAPK	Mitogen-Activated Protein Kinase
MASLD	Metabolic Dysfunction-Associated Steatotic Liver Disease
MedDiet	Mediterranean Diet
MetS	Metabolic Syndrome
MGBA	Microbiota–Gut–Brain Axis
NF-Κb	Nuclear Factor Κb
NO	Nitric Oxide
PIK3K	Phosphatidylinositol 3-Kinase
PMOS	Polyendocrine Metabolic Ovarian Syndrome
PUFAs	Polyunsaturated Fatty Acids
ROS	Reactive Oxygen Species
SCFA	Short-Chain Fatty Acid
T2DM	Type 2 Diabetes Mellitus
TMAO	Trimethylamine N-Oxide
TNF-α	Tumor Necrosis Factor-Alpha
VLCKD	Very-Low-Calorie Ketogenic Diet
WHO	World Health Organization
